# Exploring the Role of Epicardial Adipose Tissue in Coronary Artery Disease From the Difference of Gene Expression

**DOI:** 10.3389/fphys.2021.605811

**Published:** 2021-03-30

**Authors:** Qian-Chen Wang, Zhen-Yu Wang, Qian Xu, Ruo-Bing Li, Guo-Gang Zhang, Rui-Zheng Shi

**Affiliations:** ^1^Department of Cardiovascular Medicine, Xiangya Hospital, Central South University, Changsha, China; ^2^Department of Cardiovascular Medicine, The Second Xiangya Hospital, Central South University, Changsha, China; ^3^Department of Cardiovascular Surgery, Xiangya Hospital, Central South University, Changsha, China

**Keywords:** epicardial adipose tissue, coronary artery disease, gene expression profiles, bioinformatics analysis, mRNA

## Abstract

**Objectives:**

Epicardial adipose tissue (EAT) is closely adjacent to the coronary arteries and myocardium, its role as an endocrine organ to affect the pathophysiological processes of the coronary arteries and myocardium has been increasingly recognized. However, the specific gene expression profiles of EAT in coronary artery disease (CAD) has not been well characterized. Our aim was to investigate the role of EAT in CAD at the gene level.

**Methods:**

Here, we compared the histological and gene expression difference of EAT between CAD and non-CAD. We investigated the gene expression profiles in the EAT of patients with CAD through the high-throughput RNA sequencing. We performed bioinformatics analysis such as functional enrichment analysis and protein-protein interaction network construction to obtain and verify the hub differentially expressed genes (DEGs) in the EAT of CAD.

**Results:**

Our results showed that the size of epicardial adipocytes in the CAD group was larger than in the control group. Our findings on the EAT gene expression profiles of CAD showed a total of 747 DEGs (fold change >2, *p* value <0.05). The enrichment analysis of DEGs showed that more pro-inflammatory and immunological genes and pathways were involved in CAD. Ten hub DEGs (*GNG3*, *MCHR1*, *BDKRB1*, *MCHR2*, *CXCL8*, *CXCR5*, *CCR8*, *CCL4L1*, *TAS2R10*, and *TAS2R41*) were identified.

**Conclusion:**

Epicardial adipose tissue in CAD shows unique gene expression profiles and may act as key regulators in the CAD pathological process.

## Introduction

Obesity, hypertension, type 2 diabetes, hyperlipidemia, and other pathogenic factors are all associated with coronary artery disease (Gensini et al., 1998; Zengin et al., 2015; Messerli et al., 2019). Recent studies have found that in addition to the accumulation of peripheral fat, the increase in local epicardial fat is also one of the important risk factors for coronary artery disease (CAD) (Franssens et al., 2017). In addition to storing fat, adipose tissue has been increasingly recognized as an endocrine organ, particularly because it is related to glucose and lipid homeostasis (Chong et al., 2017; Wong et al., 2017; Gruzdeva et al., 2019). Excessive calorie intake will trigger chronic inflammatory changes in adipose tissue (Sekimoto et al., 2015), and the link between adipose tissue inflammation and CAD has been gradually attracting attention.

Adipose tissue mainly includes two types, white adipose tissue (WAT) and brown adipose tissue (BAT). In terms of morphology and distribution, WAT containing unilocular lipid droplets is the main body fat, which contains less cytoplasm and mitochondria. BAT containing polylocular lipid droplets is mainly distributed in the inside of the scapula or around the kidney. Its mitochondria express abundant uncoupling protein 1 (UCP1), and are rich in blood vessels and innervation. Cold and β3 receptor stimulation can trigger the yielding of UCP1-positive cells in WAT, which are similar in shape to BAT adipocytes, with multilocular lipid droplets and abundant mitochondria, and are named as “browning” adipocytes (Alipoor et al., 2020; Reinisch et al., 2020). Under physiological conditions, Epicardial adipose tissue (EAT) belongs to WAT, but under pathological conditions, UCP1 expression increases in EAT which then presents “browning” changes (Chechi et al., 2017). Clinical studies have found that EAT deposition is positively correlated with increased metabolic abnormalities and CAD (Milanese et al., 2020).

Epicardial adipose tissue covers almost all coronary arteries anatomically (Iacobellis, 2015). By wrapping around and directly contacting the coronary arteries without fascia separation, EAT is able to affect the pathophysiological process of coronary arteries. EAT is mainly located in the atrioventricular and ventricular sulcus. It is regarded as a unique adipose depot with unique metabolic characteristics, which has a higher ability to absorb and release fatty acid (FFA) than other visceral fats and is an effective source of FFA that fuels the energy-intensive myocardial tissue (Kankaanpää et al., 2006; Pezeshkian and Mahtabipour, 2013). Many studies have shown that CAD patients have more expressions and pro-inflammatory cytokines production in the EAT than in the subcutaneous adipose tissue (SAT) (Vacca et al., 2016; Mráz et al., 2019). The EAT in CAD patients expresses pro-inflammatory adipokines and is accompanied by the infiltration of various immune cells (Nomura et al., 2020). However, most studies only compare the difference between EAT and SAT in an individual with CAD and lack the comparison with the EAT in subjects with normal coronary arteries. Therefore, it is difficult to estimate the scale of the EAT pro-inflammatory effect on coronary arteries in CAD.

In this study, we first directly compared the gene expression in the EAT of subjects with and without CAD through RNA sequencing (RNA-Seq), and obtained the gene expression profiles of EAT in CAD, and then performed bioinformatics analysis such as functional enrichment analysis and protein-protein interaction (PPI) network construction, etc., and obtained and verified differentially expressed genes with research value.

## Materials and Methods

### Study Population

The study subjects were 11 patients with CAD who underwent coronary artery bypass surgery and 11 patients without CAD who underwent valve replacement in the Department of Cardiovascular Surgery of Xiangya Hospital of Central South University. The selective and exclusive criteria were as follows: (1) CAD group were patients with CAD who underwent coronary artery bypass surgery (confirmed by coronary angiography); (2) Control group (CTRL group) were patients with valvular disease who matched the age, gender, and body mass index (BMI) of the CAD group and underwent valve replacement with no evidence of CAD syndrome and negative findings in coronary angiography; (3) The main exclusive criteria included a previous PCI history, history of diabetes mellitus, previous thoracotomy history, tumor, severe infection, and autoimmune diseases. We selected five pairs from the two groups matched in age, gender, BMI, and abdominal circumference (AC), and RNA-seq was performed on the selected pairs. All patients underwent a preoperative risk assessment and signed informed consent forms. This study was approved by the Ethics Committee of Xiangya Hospital of Central South University. Trial registration: Chinese Clinical Trial Registry, No. ChiCTR1900024782.

### Sample Collection

Before starting the extracorporeal circulation, the samples of EAT were taken from uninjured areas near the anterior descending coronary artery or along the atrioventricular sulcus near the right coronary artery. The samples were quickly frozen with liquid nitrogen for molecular biology experiments and stored at -80°C.

### Histology

The EAT samples were fixed in 4% paraformaldehyde, embedded in paraffin, and cut into 4 μm thick slices. The size of the adipocyte was determined by calculating the Feret’s diameter of the H&E stained slices (Lin et al., 2013). For each slice, we used the Image-Pro 6.2 software to check five fields of view and calculated the average.

### RNA Sequencing

Total RNA was extracted from the samples using TRIzol. RNase H method was used to remove rRNA. After RNA fragmentation, cDNA synthesis, A-Tailing Mix, and adapters were added to establish a library. An Agilent 2100 Bioanalyzer (Agilent DNA 1000 Reagents) was used to detect the inserted fragments of the library, and HiSeq4000 (BGI-Shenzhen, China) was used to sequence the library qualified for detection.

### Functional Enrichment Analysis of DEGs

The database for annotation, visualization, and integrated discovery (DAVID 6.8^1^) (Huang da et al., 2009) was used to analyze the functional enrichment of key differentially expressed genes (DEGs) in the GO Terms and KEGG Pathways. The threshold was *p* < 0.05.

### PPI Network Construction and Analysis

Studying the interaction between the encoded proteins will help us to discover the core regulatory genes. Thus, in order to explore the DEGs with a research value, a search tool (STRING 10.5^2^) (Szklarczyk et al., 2019) was used to establish a PPI network, and Cytoscape (Shannon et al., 2003) was used to plot the network. Interactions with a score >0.4 were set as the cutoff point. The most important modules in the PPI network were identified using MCODE in Cytoscape, which clustered a given network through topology. The threshold was set as: MCODE scores > 5, degree cut-off = 2, node score cut-off = 0.2, max depth = 100, and *k*-score = 2. Subsequently, the maximal clique centrality (MCC) algorithm of CytoHubba was used to explore the hub genes of the PPI network.

### Real-Time Quantitative PCR

Total RNA was extracted from the samples using TRIzol (Takara) according to the manufacturer’s instruction. We used the cDNA synthesis kit (Takara) to conduct reverse transcription. Real-time quantitative PCR (RT-qPCR) was performed by the FastStart Universal SYBR Green Master (ROX) (Takara). 2^–ΔΔ*Ct*^ relative quantification method was used for relative expression. β-actin was used as the internal reference (Sangon Biotech). The primer sequences were as following: *UCP1* forward CGACGTCCAGTGTTATTAGGTA, *UCP1* reverse GTAGAGTTTCATCCGCCCTTC, *GNG3* forward CGGTGAACAGCACTATGAGTAT, *GNG3* reverse TCACAGTA AGTCATCAGGTCTG, *MCHR1* forward CGCTTGGTCC TGTCGGTGAAG, *MCHR1* reverse GCCTTTGCTTTCTG TCCTCTCCTC, *BDKRB1* forward CTTTTTGTCCTGTTGG TCTTCC, *BDKRB1* reverse CTGATGAACAAATTGGCCTTGA, *MCHR2* forward AACCTGGCTGTGGCTGATTTGG, *MCHR2* reverse GGGATGTGATGATGGTGCAGAGAG, *CXCL8* forward AACTGAGAGTGATTGAGAGTGG, *CXCL8* reverse ATGA ATTCTCAGCCCTCTTCAA, *CXCR5* forward CGGCAG ACACGCAGTTCCAC, *CXCR5* reverse ACGGCAAAGGGCAA GATGAAGAC, *CCR8* forward TGGCCCTGTCTGACCT GCTTT, *CCR8* reverse GGCATAAGTCAGCTGTTGGCT, *CCL4L1* forward CTCAGTATCAGCACAGGACACAGC, *CCL 4L1* reverse AGAGACAGGACAGTCACGCAGAG, *TAS2R10* forward AGTGTTTGGGGTTTTGGGGAATGG, *TAS2R10* reverse AGCCGGTGAGAATAAAGCCAATCG, *TAS2R41* forward ACTCAGCCACCTTCTGGTTTTGC, and *TAS2R41* reverse ATCAGGACAGAGCCCAACAGGAG.

### Statistical Analysis

The statistical analysis was performed by SPSS25.0 (IBM). All values were expressed as mean ± SD. Statistically significant differences were assessed by ANOVA and Chi-squared test for comparisons between two groups. Two-tailed *p* value <0.05 was considered statistically significant.

## Results

### Study Population

The clinical characteristics of the study subjects are listed in Table 1. There was no significant difference in age, gender, body mass index (BMI), abdominal circumference (AC), comorbidities, and plasma lipid levels between the two groups. The average age of the control subjects was 56.0 ± 8.0 years and the CAD subjects was 58.8 ± 7.0 years (*p* = 0.392). Therefore, our research groups were well matched except for the presence of CAD.

**TABLE 1:** Patient characteristics.

	CTRL (*n* = 11)	CAD (*n* = 11)	*p* values
Age (years)	56.0 ± 8.0	58.8 ± 7.0	0.392
Sex, male (%)	6 (54.5)	5 (45.5)	0.748
BMI (Kg/m^2^)	22.5 ± 2.4	23.8 ± 2.1	0.197
AC (cm)	86.1 ± 10.3	87.2 ± 6.6	0.779
**Complications**			
Diabetes mellitus (%)	0 (0.0)	0 (0.0)	1.000
Hypertension (%)	3 (27.2)	5 (45.5)	0.478
**CAD**			
1-Vessel disease	0 (0.0)	1 (9.1)	
2-Vessel disease	0 (0.0)	4 (36.4)	
3-Vessel disease	0 (0.0)	6 (54.5)	
Gensini score	3.6 ± 1.4	88.4 ± 43.7	<0.001
**Laboratory measurements**			
WBC (×10^9^/L)	5.9 ± 2.7	6.5 ± 1.4	0.513
TG (mmol/L)	1.6 (0.6–2.6)	1.6 (1.2–2.0)	0.997
TC (mmol/L)	4.2 ± 0.8	4.6 ± 1.7	0.411
LDL-c (mmol/L)	2.6 ± 0.5	2.9 ± 1.3	0.472
**Left heart function**			
EF (%)	55.6 ± 9.9	58.0 ± 12.6	0.636

*Data are presented as mean ± SD or the number (%) of patients. BMI, body mass index; AC, abdominal circumference; WBC, white blood cell; TGs, triglycerides; TC, total cholesterol; LDL-c, low-density lipoprotein cholesterol; EF, ejection fraction.*

### Comparison of EAT in Histology

**FIGURE 1:**
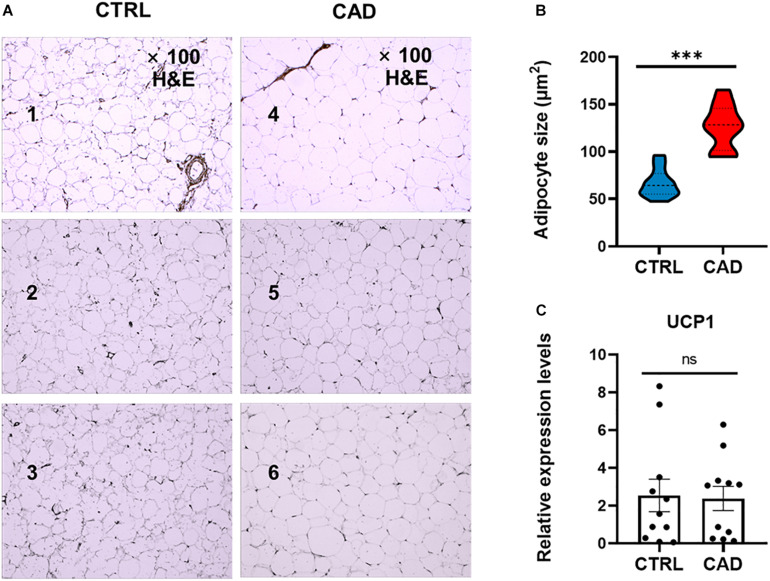
Comparison of EAT in histology. **(A)** H&E staining of epicardial adipose tissue (EAT) in the control and coronary artery disease (CAD) groups (100× magnification). Numbers 1–3 were EAT samples of three control subjects, while numbers 4–6 were EAT samples of three CAD patients. **(B)** The mean adipocyte size was larger in CAD group (***p < 0.001). **(C)** Expression levels of uncoupling protein 1 (UCP1) in the CTRL and CAD group (ns, *p* > 0.05).

The EAT samples of both groups were mainly unilocular white adipocytes (Figure 1A). The sizes of EAT adipocytes in the CAD group were significantly larger than those in the control group (126.59 ± 23.27 μm^2^ and 66.45 ± 14.90 μm^2^, *p* < 0.001) (Figure 1B). Then, we compared the “browning” of fat in each group by detecting the expression of UCP1 (Figure 1C) and found that the level UCP1 was not different in the two groups (*p* > 0.05).

### EAT Gene Expression Profiles in CAD

**FIGURE 2:**
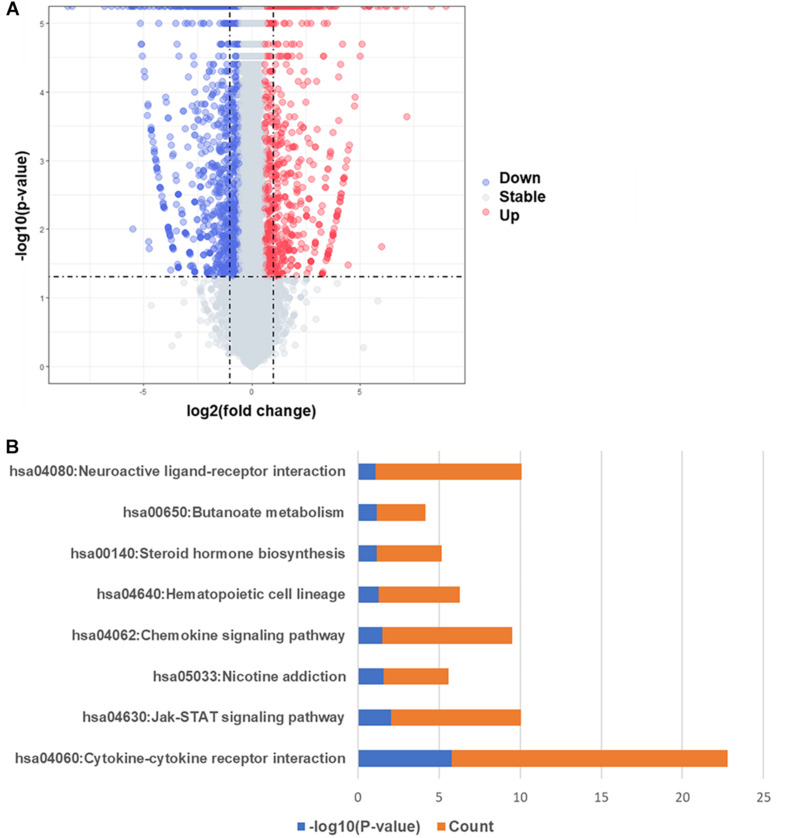
Differentially expressed genes and KEGG pathway enrichment analysis. **(A)** Volcano map of differentially expressed genes, red spots represent up-regulated genes and blue spots represent down-regulated genes. **(B)** KEGG pathways significantly associated with the upregulated genes.

In order to compare the difference of gene expression in EAT between the CAD and non-CAD groups, we used the high-throughput RNA sequencing. The thresholds of differentially expressed genes (DEGs) were fold change > 2 and adjusted *p* value < 0.05. In total, there were 747 DEGs, of which, 301 were significantly up-regulated in the CAD group and 446 genes were significantly down-regulated (Figure 2A). Enrichment analysis of 301 up-regulated differential genes showed that the KEGG pathways were significantly enriched in cytokine-cytokine receptor interaction, jak-STAT signaling pathway, nicotine addiction, chemokine signaling pathway, hematopoietic cell lineage, steroid hormone biosynthesis, butanoate metabolism, and neuroactive ligand-receptor interaction (Figure 2B). The top eight GO analysis results were listed in Table 2. The biological processes (BP) of GO analysis showed that genes were mainly enriched in epidermis development, cytoskeleton organization, cellular calcium ion homeostasis, chemokine-mediated signaling pathway, chemotaxis, neutrophil chemotaxis, steroid metabolic process, and lymphocyte chemotaxis.

**TABLE 2:** The significant GO enriched analysis of differentially expressed genes in CAD.

Category	Term	Count	*p*-value	Genes
GO_BP	GO:0008544	11	0.000000129	*PTHLH, EVPLL, ELF3, CST6, KRT5, KRT16, POU2F3, KRT15, SPRR2F, LAMC2*,and *EDAR*
	Epidermis development			
	GO:0007010	13	0.00000145	*BUB1B-PAK6, ARC, CCL2, BLK, CTNNA2, CCL24, CCL11, KISS1, DES*, and *KRT5*
	Cytoskeleton organization			
	GO:0006874	8	0.0000854	*CCL11, CCL2, SLC8A2, TRPM8, GRIN1, CHRFAM7A, ATP13A5*, and *ATP13A4*
	Cellular calcium ion homeostasis			
	GO:0070098	7	0.001093	*CCL24, CCL11, CCR8, CCL2, CXCR5, CXCL8*, and *CCL4L2*
	Chemokine-mediated signaling pathway			
	GO:0006935	8	0.004041	*CCL24, CCL11, CCR8, CCL2, CXCR5, CXCL8, SERPIND1*, and *FOSL1*
	Chemotaxis			
	GO:0030593	6	0.004568	*CCL24, CCL11, CCL2, EDN2, CXCL8*, and *CCL4L2*
	Neutrophil chemotaxis			
	GO:0008202	5	0.005327	*AKR1C4, CYP3A7, CYP1A1, AKR1B10*, and *SULT1A3*
	Steroid metabolic process			
	GO:0048247	4	0.010621	*CCL24, CCL11, CCL2*, and *CCL4L2*
	Lymphocyte chemotaxis			
GO_CC	GO:0005615	52	1.95E-08	*HMSD, NOG, LYPD3, KERA, MASP1, EDN2, FAM3B, IL13, DLK1, IL11*,…
	Extracellular space			
	GO:0005576	50	0.00000209	*LYPD2, NOG, KERA, CNDP1, MASP1, EDN2, FAM3B, BCAN, IL13, IL11*,…
	Extracellular region			
	GO:0005882	9	0.0000576	*KRT72, KRT6A, DES, KRT5, KRT16, KRT15, KRT7, BFSP2*, and *KRT86*
	Intermediate filament			
	GO:0005886	88	0.00618	*LYPD2, CYP24A1, MCHR1, MCHR2, SYT4, OR4F21, LYPD5, PRSS41, SLC6A4*, and *SYT3*,
	Plasma membrane			
	GO:0045095	6	0.025111	*KRT72, KRT6A, KRT5, KRT7, KRT86*, and *KRT71*
	Keratin filament			
	GO:0005887	34	0.028064	*MCHR1, MCHR2, SLC6A4, AQP4, MFSD2A, BDKRB1, AQP6, GCGR, GPRC5A, CXCR5*,…
	Integral component of plasma membrane			
	GO:0005578	10	0.034652	*WNT10A, COL9A2, CPA6, ZP2, KERA, IMPG1, DGCR6, ENAM, BCAN*, and *MUC4*
	Proteinaceous extracellular matrix			
	GO:0016021	100	0.045498	*LYPD3, SYT4, SERTM1, OR4F21, B3GALT5, SLC9A2, SYT3, SLC6A4, AQP4, OR1E1*,…
	Integral component of membrane			
GO_MF	GO:0005200	9	0.000397	*KRT6A, DES, KRT5, KRT16, KRT15, TUBAL3, BFSP2, NEFH*, and *CTNNA2*
	Structural constituent of cytoskeleton			
	GO:0005198	13	0.000673	*KRT72, EVPLL, KRT6A, DES, KRT5, KRT16, KRT15, KRT7, NEFH, SPRR2F*,…
	Structural molecule activity			
	GO:0008009	5	0.007812	*CCL24, CCL11, CCL2, CXCL8*, and *CCL4L2*
	Chemokine activity			
	GO:0005544	5	0.01399	*SYT4, SYT3, SYTL5, SYT13*, and *PLA2G4B*
	Calcium-dependent phospholipid binding			
	GO:0005509	20	0.022734	*S100A5, MASP1, SYT4, GRIN1, SYT3, CAPN9, RCVRN, CDH2, DLK1, CLGN*,…
	Calcium ion binding			
	GO:0005125	8	0.023818	*LIF, CSF3, FAM3B, LEFTY2, IL13, IL24, IL11*, and *IL20*
	Cytokine activity			
	GO:0005328	3	0.033172	*SLC6A4, SLC6A15*, and *SLC6A17*
	Neurotransmitter: sodium symporter activity			

### PPI Network Construction and Hub Genes Verification

**FIGURE 3:**
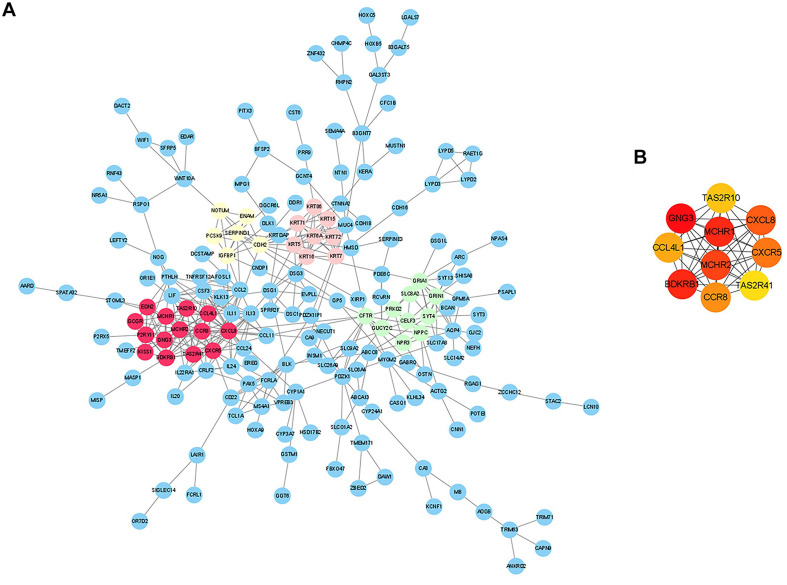
Analysis of protein-protein interaction (PPI) network of CAD-related differentially expressed genes (DEGs) in EAT. **(A)** Edges represented protein-protein associations which were meant to be specific and meaningful. The colored nodes (red, green, pink, and yellow) represented the top four modules in the PPI network. **(B)** Top 10 hub genes identified by CytoHubba in Cytoscape.

**FIGURE 4:**
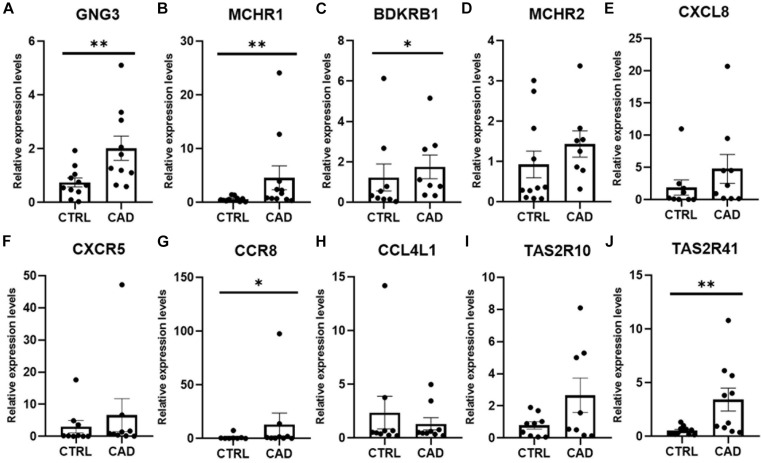
Real-time quantitative PCR (RT-qPCR) verification of 10 hub genes in EAT of CAD. **(A)** GNG3 expression was significantly increased in the CAD group. **(B)** MCHR1 expression was significantly higher in the CAD group. **(C)** BDKRB1 expression was greater in the CAD group. **(D)** MCHR2 was increased in the CAD group, although the difference was not statistically significant. **(E)** CXCL8 expression was not statistically different between the two groups. **(F)** CXCR5 expression was higher in the CAD group, although the difference was not statistically significant. **(G)** CCR8 expression was significantly higher in the CAD group. **(H)** CCL4L1 expression was not different in the two groups. **(I)** TAS2R10 expression was increased in the CAD group, although the difference was not statistically significant. **(J)** TAS2R41 expression was significantly increased in EAT of CAD. *p < 0.05, **p < 0.01. In each column, error bars show the mean and standard deviation per group.

To further identify the key genes with research value, 301 up-regulated DEGs were submitted to the STRING database to predict the interaction between proteins. DEGs’ PPI network was constructed with a comprehensive score greater than 0.4 (Figure 3A), and the most important module was obtained using MCODE in Cytoscape. The first module included 14 nodes and 67 edges; the second module included eight nodes and 28 edges; and the third module included six nodes and 15 edges. The hub genes were selected from the PPI network using the maximal clique centrality (MCC) algorithm of CytoHubba (Figure 3B). The top 10 hub genes identified by MCC were *GNG3, MCHR1, BDKRB1, MCHR2, CXCL8, CXCR5, CCR8, CCL4L1, TAS2R10*, and *TAS2R41.* RT-qPCR was performed using the total RNA extracted from 11 pairs of EAT to verify the expression levels of the hub genes. The results of the RT-qPCR showed significant changes in the expression levels of hub genes such as *GNG3, MCHR1, BDKRB1, CCR8*, and *TAS2R41* in Figure 4 (*p* value < 0.05).

## Discussion

Epicardial adipose tissue directly contacts with the coronary arteries without fascia isolation, which provides an important anatomical basis for the interaction between them. EAT can be used as a target for drugs targeting adipose tissue. New hypoglycemic drugs, such as glucagon-like peptide-1 receptor (GLP-1R) agonists and glucose-sodium co-transporter 2 inhibitors (SGLT2i) (Bouchi et al., 2017; Iacobellis et al., 2017), and lipid-lowering drugs such as atorvastatin (Raggi et al., 2019), have been shown to directly target the EAT and reduce its volume. GLP-1R’s regulation of EAT promotes fatty acid β-oxidation and white-to-brown adipocyte differentiation, promoting favorable metabolic changes. Clinical studies have shown that EAT is remarkably correlated with the presence of CAD (Le Jemtel et al., 2019; Liu et al., 2019; Nappi et al., 2019). Previous studies have focused on comparing the difference between EAT and SAT. Different from previous studies, we directly compared the differences in EAT gene expression profiles between CAD and non-CAD subjects, providing novel information to describe the genomic characteristics of EAT in healthy and diseased subjects. Then, we provided evidence that, compared with the control group, EAT in CAD was characterized by enhanced inflammatory genes, metabolic remodeling, and fat remodeling. These results might help us discover the key genes and therapeutic targets in CAD, which might provide a more theoretical basis for exploring the pathogenesis of CAD.

Our research confirmed the findings of some previous studies and suggested some potentially novel discoveries. Our results showed that the size of epicardial adipocytes in the CAD group was larger than the control group, which was consistent with the results of previous studies (Vianello et al., 2016). However, we found that the “browning” marker UCP1 was not significantly different in the two groups. Our findings of the EAT gene expression profiles of CAD showed a total of 747 DEGs, of which, 301 DEGs were up-regulated and 446 DEGs were down-regulated. It was notable that the enrichment analysis of DEGs showed that more pro-inflammatory and immunological genes and pathways were involved in CAD, including chemokine-mediated signaling pathway, chemotaxis, neutrophil chemotaxis, lymphocyte chemotaxis, chemokine activity, cytokine activity, cytokine-cytokine receptor interaction, and chemokine signaling pathway. This suggested that inflammatory factors and immune cell activation might play an important role in the regulation of CAD. We constructed the DEG’s PPI network in which three key modules and 10 central genes were explored, including *GNG3, MCHR1, BDKRB1, MCHR2, CXCL8, CXCR5, CCR8, CCL4L1, TAS2R10*, and *TAS2R41*. In addition, we used RT-qPCR to further verify the results.

A meta-analysis focusing on the transcriptome assessment of EAT in CAD patients confirmed the activation of inflammatory, immune, and metabolic pathways in CAD–EAT, and highlighted interleukin-6 (IL-6) and tumor protein p53 (TP53) as core genes (Maghbooli and Hossein-Nezhad, 2015). McKenney et al. (2014) demonstrated in a porcine model that the resection of local EAT can prevent CAD progression. These results suggest that EAT dysfunction may lead to changes in inflammation and metabolic microenvironment, thereby affecting vascular homeostasis, and may trigger coronary atherosclerosis. We found that the salient features of EAT in CAD include an enhanced communication between the inflammatory cells and chemokine signaling (*CXCL8*, *CXCR5*, *CCR8*, and *CCL4L1*). Studies have confirmed that many CC and CXC chemokines are involved in cardiovascular diseases (Frangogiannis and Entman, 2005; Frangogiannis, 2014). Cytokines in the EAT of CAD are also gradually attracting attention. Immunocyte like monocytes and neutrophils infiltrate the intima and activate endothelial cells, which induce the differentiation of monocytes into macrophages and formation of foam cells. CXC chemokines like *CXCL8* was reported to control neutrophil infiltration (Dyer et al., 2014) and CXCR5 + T cells was found to contribute to inflammatory reactions in CAD (Ding et al., 2017). *CCR8* can recruit IL-5 + T(H)2 cells (Islam et al., 2011) and act as a driver of atherosclerosis (Gast et al., 2019). *CCL4L1* may play a role in aortic aneurysm (Gäbel et al., 2017) though its involvement in CAD has yet to be clarified. Overall, our findings indicate that the immunological function of *CXCL8*, *CXCR5*, *CCR8*, and *CCL4L1* in the pathogenesis of EAT in CAD may be the focus of future investigations.

The other six hub genes had been rarely studied in the field of cardiovascular research. *GNG3* has not been reported in CAD. However, Schwindinger et al. (2004) reported that the mice which were lacking *GNG3* had a significantly reduced weight. Balber et al. (2019) reported that *MCHR1* expression was increased in BAT, and the use of *MCHR1* antagonists in rodents was able to reduce adipogenesis. It is well known that obesity is a risk factor of CAD and the findings of these genes may suggest that they may be indirectly involved in CAD pathogenesis. The discovery of the melanin-concentrating hormone (MCH) and its receptors (*MCHR1* and *MCHR2*) secreted by the hypothalamus may provide a new target for the research on the mechanism of obesity and therefore may contribute to the prevention of CAD. Schulze-Topphoff et al. (2009) provided evidence that Bdkrb1 may be a therapeutic target for chronic inflammation. TAS2R10 and *TAS2R41* can stimulate the secretion of ghrelin in gastric fundic cells (Wang et al., 2019), yet they have not been studied in the cardiovascular system. These newly discovered genes in EAT that we have discovered may provide new targets for CAD research.

However, there are some limitations in our study. The main limitation of the study could be the ethical (quantity of total adipose tissue) limitation. Many CAD patients undergoing coronary artery bypass grafting have diabetes, which our study needs to exclude, making it even more difficult to obtain the samples. In addition, the present study stopped short of validating and further exploring the related mechanisms because of the limitation of culturing human epicardial adipocytes. Further studies are required to gain insight into the pathogenesis.

In summary, in this study, we explored the gene expression profiles of EAT in CAD. We found that EAT may participate in CAD through key genes including *GNG3, MCHR1, BDKRB1, MCHR2, CXCL8, CXCR5, CCR8, CCL4L1, TAS2R10*, and *TAS2R41*, and some novel pathways, including cytokine-cytokine receptor interaction, jak-STAT signaling pathway, nicotine addiction, chemokine signaling pathway, hematopoietic cell lineage, steroid hormone biosynthesis, butanoate metabolism, and neuroactive ligand-receptor interaction. These results may help us explore the role of EAT in CAD from a new and deeper perspective.

## Data Availability Statement

The datasets presented in this study can be found in online repositories. The names of the repository/repositories and accession number(s) can be found below: https://figshare.com/ and https://doi.org/10.6084/m9.figshare.12826109.v1.

## Ethics Statement

The studies involving human participants were reviewed and approved by the Ethics Committee of Xiangya Hospital of Central South University. The patients/participants provided their written informed consent to participate in this study.

## Author Contributions

Q-CW and Z-YW designed the protocols and performed the experiments. QX and R-BL included the patients and collected the samples. G-GZ and R-ZS did the formal analysis. All the authors have contributed to the writing of the manuscript.

## Conflict of Interest

The authors declare that the research was conducted in the absence of any commercial or financial relationships that could be construed as a potential conflict of interest.

## Footnotes

1 http://david.ncifcrf.gov

2 http://string-db.org
